# Bicarbonate buffered peritoneal dialysis fluid upregulates angiopoietin-1 and promotes vessel maturation

**DOI:** 10.1371/journal.pone.0189903

**Published:** 2017-12-18

**Authors:** Gwendolyn Eich, Maria Bartosova, Christian Tischer, Tanja Tamara Wlodkowski, Betti Schaefer, Sebastian Pichl, Nicole Kraewer, Bruno Ranchin, Karel Vondrak, Max Christoph Liebau, Thilo Hackert, Claus Peter Schmitt

**Affiliations:** 1 Center for Pediatric and Adolescent Medicine, University Hospital Heidelberg, Heidelberg, Germany; 2 The European Molecular Biology Laboratory, Heidelberg, Germany; 3 Service de Néphrologie Pédiatrique, Hôpital Femme Mère Enfant, Hospices Civils de Lyon, France; 4 Department of Pediatrics, University Hospital Motol, Prague, Czech Republic; 5 Pediatric Nephrology, Department of Pediatrics and Center for Molecular Medicine, University Hospital of Cologne, Cologne, Germany; 6 Department of Surgery, University Hospital Heidelberg, Heidelberg, Germany; Seoul National University College of Pharmacy, REPUBLIC OF KOREA

## Abstract

**Background:**

Ultrafiltration decline is a progressive issue for patients on chronic peritoneal dialysis (PD) and can be caused by peritoneal angiogenesis induced by PD fluids. A recent pediatric trial suggests better preservation of ultrafiltration with bicarbonate versus lactate buffered fluid; underlying molecular mechanisms are unknown.

**Methods:**

Angiogenic cytokine profile, tube formation capacity and Receptor Tyrosine Kinase translocation were assessed in primary human umbilical vein endothelial cells following incubation with bicarbonate (BPDF) and lactate buffered (LPDF), pH neutral PD fluid with low glucose degradation product content and lactate buffered, acidic PD fluid with high glucose degradation product content (CPDF). Peritoneal biopsies from age-, PD-vintage- and dialytic glucose exposure matched, peritonitis-free children on chronic PD underwent automated histomorphometry and immunohistochemistry.

**Results:**

In endothelial cells angiopoietin-1 mRNA and protein abundance increased 200% upon incubation with BPDF, but decreased by 70% with LPDF as compared to medium control; angiopoietin-2 remained unchanged. Angiopoietin-1/Angiopoietin-2 protein ratio was 15 and 3-fold increased with BPDF compared to LPDF and medium. Time-lapse microscopy with automated network analysis demonstrated less endothelial cell tube formation with BPDF compared to LPDF and CPDF incubation. Receptor Tyrosine Kinase translocated to the cell membrane in BPDF but not in LPDF or CPDF incubated endothelial cells. In children dialyzed with BPDF peritoneal vessels were larger and angiopoietin-1 abundance in CD31 positive endothelium higher compared to children treated with LPDF.

**Conclusion:**

Bicarbonate buffered PD fluid promotes vessel maturation via upregulation of angiopoietin-1 *in vitro* and in children on dialysis. Our findings suggest a molecular mechanism for the observed superior preservation of ultrafiltration capacity with bicarbonate buffered PD fluid with low glucose degradation product content.

## Introduction

Peritoneal dialysis (PD) efficacy depends on the peritoneal membrane integrity and transport function, which deteriorates with chronic PD. PD fluids are composed of non-physiological concentrations of glucose, lactate and glucose degradation products at an acidic pH. Peritoneal membrane transformation comprises mesothelial cell loss, progressive fibrosis and angiogenesis [1]. Overshooting angiogenesis increases peritoneal vessel number, accelerates glucose uptake from the dialysate into the circulation and thus dissipation of the osmotic gradient required for ultrafiltration. To maintain adequate ultrafiltration, increasing dialysate concentrations of glucose are prescribed, further promoting angiogenesis in a vicious cycle, which ultimately leads to PD failure [2].

Separation of glucose at a very low pH from the buffer compound during heat sterilization and storage substantially reduces glucose degradation product formation and allows for a neutral pH of the PD fluid after mixture. Clinical trials suggest improved biocompatibility based on effluent markers CA125 [3], hyaluronan [4], vascular endothelial growth factor (VEGF) and interleukin-6 [5]. A recent trial demonstrated lower ultrafiltration rates during the first 6 months of PD with fluids with low glucose degradation product content but better preservation of long term PD function as compared to the acidic PD fluids with high glucose degradation product content [6]. A randomized trial comparing two neutral pH PD fluids with low glucose degradation product content containing either lactate or bicarbonate buffer over 10 months yielded better preservation of ultrafiltration capacity with the bicarbonate PD fluid [7].

*In vitro*, lactate exerts significant mesothelial cell toxicity, even at neutral pH [8]. Bicarbonate buffered PD fluid with low glucose degradation product content induces less mesothelial gene regulation [9], less oncosis [10] and up-regulates aquaporin-1 dependent mesothelial cell migration [11]. In rats, pH adjusted lactate fluid induces more mesenterial angiogenesis than the respective bicarbonate buffered fluid, independent of the presence of glucose [12]. The molecular mechanisms remain elusive. We now analyzed the effect of lactate and bicarbonate buffered PD fluids with low glucose degradation product content on endothelial cell angiogenic cytokine profile, and angiopoietin-1 induced Receptor Tyrosine Kinase translocation and PD fluid dependent endothelial cell vessel formation capacity *in vitro*. We then validated our *in vitro* findings in peritoneal tissue specimens of age, PD vintage and glucose exposure matched children on chronic PD with low glucose degradation product content PD fluid containing lactate and bicarbonate buffer, respectively.

## Materials and methods

### Cell culture

Human umbilical vein endothelial cells were purchased commercially (PromoCell) and kept in endothelial cell growth medium with supplement, antibiotics and 10% fetal calf serum. Experiments were performed in fetal calf serum and supplement free medium after 24h starvation. Isolation of human peritoneal mesothelial cells from omental tissue of patients undergoing abdominal surgery was approved by the Ethic committee of the Medical Faculty, University of Heidelberg. Informed written consent was obtained from the patients. Human peritoneal mesothelial cells were isolated from four non-uremic patients and propagated as previously described [13]. Human endothelial and human peritoneal mesothelial cells were incubated with acidic, (pH 5.5), lactate buffered PD fluid (CPDF; CAPD^®^),with high glucose degradation product content and two fluids with neutral pH, low glucose degradation product content buffered either with lactate (LPDF; Balance^®^) or bicarbonate (BPDF; BicaVera^®^) and medium. Glucose concentration was 2.3%.

### Quantitative PCR

Ribonucleic acid (RNA) isolation was performed with cells plated at a density of 2.5 × 10^5^ cells/well in six-well plates and grown until 70–80% confluence. RNA was isolated with the RNAeasy MiniKit (Qiagen, Hilden, Germany) according to the manufacturer´s instructions, checked for integrity on an agarose gel and quantified photometrically. Quantitative Real Time PCR (qRT-PCR) was performed using the StepOnePlus Real Time PCR System by Applied Biosystems. 1μg of RNA was transcribed to cDNA using the high capacity cDNA reverse transcription kit (Applied Biosystems). The cDNA was incubated with a master mix consisting of 2xSYBR Green and the forward and reverse primers of heme oxygenase-1 (forward ACATCCAGCTCTTTGAGGAGTTG; reverse GCAGAATCTTGCACTTTGTTGCT-), angiopoietin-1 (forward TGGCTGCAAAAACTTGAGAATTAC-; reverse TTCTGGTCTGCTCTGCAGTCTG-), angiopoietin-2 (forward-AACAGGAGGCTGGTGGTTTG-; reverse-AATGCCGTTGAACTTATTTGTGTTC-), fibroblast growth factor-2 (forward-CCCTCACATCAAGCTACAACTTGA-; reverse-AAGCCAGTAATCTTCCATCTTCCTT-), Vascular Endothelial Growth Factor (VEGF-A: forward-CCATGCAGATTATGCGGATCA-; reverse-TCTTTGGTCTGCATTCACATTTGT-), hypoxia-inducible factor-1a (forward-TTGTGATGAAAGAATTACGAATTG-; reverse-GTGACTTGTCCTTTAGTAAACATATCATGAT-), matrix metalloproteinase-1 (forward-ATGCTGAAACCCTGAAGGTG-; reverse-TTGGAAGGCTTTCTCAATGG-), aquaporin-1 (forward-ATGACCTGGCTGATGGTGTGA-; reverse-CGCCTCCGGTCGGTAGTAG-) and 18S (forward-AGTTGGTGGAGCGATTTGTC-; reverse-CGGACATCTAAGGGCATCAC-) in a total reaction volume of 20 μl. Thermal cycling protocol was one minute at 95°C followed by 45 cycles à 15 seconds at 95°C and one minute at 60°C. Serial dilutions of a pooled cDNA consisting of all measured samples were used to establish a standard curve. All measurements were performed in duplicates. Relative quantity was normalized to the internal control 18S mRNA.

### Western blot

Cells were lysed with lysis buffer (containing 1mM NatriumVanadat, 1mM NatriumFluorid, 1x phosphatase inhibitor Calyculin A, 1 x complete mini; added Volume with tissue protein extraction reagent (Thermo Fisher Scientific, Waltham, USA)). Equal amount of 30 μg of total protein in each sample of cell lysate was diluted with 4x loading buffer (containing 4% ß-mercaptoethanol). Proteins were separated in a 10% polyacrylamide gel at 200 V for 45 min. The transfer of the protein onto a polyvinylidenfluoride (PVDF) membrane was performed in a Transblot Cell (Bio-Rad Laboratories, Munich, Germany) at 105 V for 90 min. The membrane was blocked with blocking buffer (3% bovine serum albumin, 5% milk) for 2 hours at room temperature followed by incubation with specific antibodies against (Angiopoietin-1, Sigma Aldrich, St. Louis, Missouri, USA, 1:250; Angiopoietin-2, Atlas Antibodies, Stockholm, Sweden, 1:1000) overnight at 4°C. The membrane was rinsed once and washed three times for 5, 10 and 15 min with 0.05% Tris-buffered saline with Tween20 (TBS-T), then incubated for one hour at room temperature with secondary antibody (anti rabbit horseradish peroxidase conjugated, 1:3000). Following three further rinsing procedures with 0.05% TBS-T, enhanced chemiluminescent signal detection was performed. Equal loading of protein was assessed by reprobing the membrane for glyceraldehyde 3-phosphate dehydrogenase (GAPDH). The signals were scanned and quantified densitometrically using Image Lab Software® (Bio-Rad, USA).

### Immunofluorescence staining

PD fluid treated human endothelial cells were methanol: acetone fixed (10 min), blocked with universal blocking reagent (30 min, 37°C) and incubated overnight with antibody against Receptor Tyrosine Kinase (4°C; R&D Systems). Secondary Cy3 antibody was added and incubation (30min at 37°C) was followed by blocking. Antibody against vascular-endothelial cadherin (VE-cadherin) (1:500, Abcam) was added for 1h followed by AlexaFluor488 antibody. Nuclei were 4′,6-Diamidin-2-phenylindol (DAPI) stained and preparations visualized by fluorescence microscopy with equal exposure times. Recombinant human angiopoietin-1 treated human endothelial cells (600ng/ml; R&D Systems) were used as positive control of Receptor Tyrosine Kinase translocation.

### Tube formation assay

**Fig 1:**
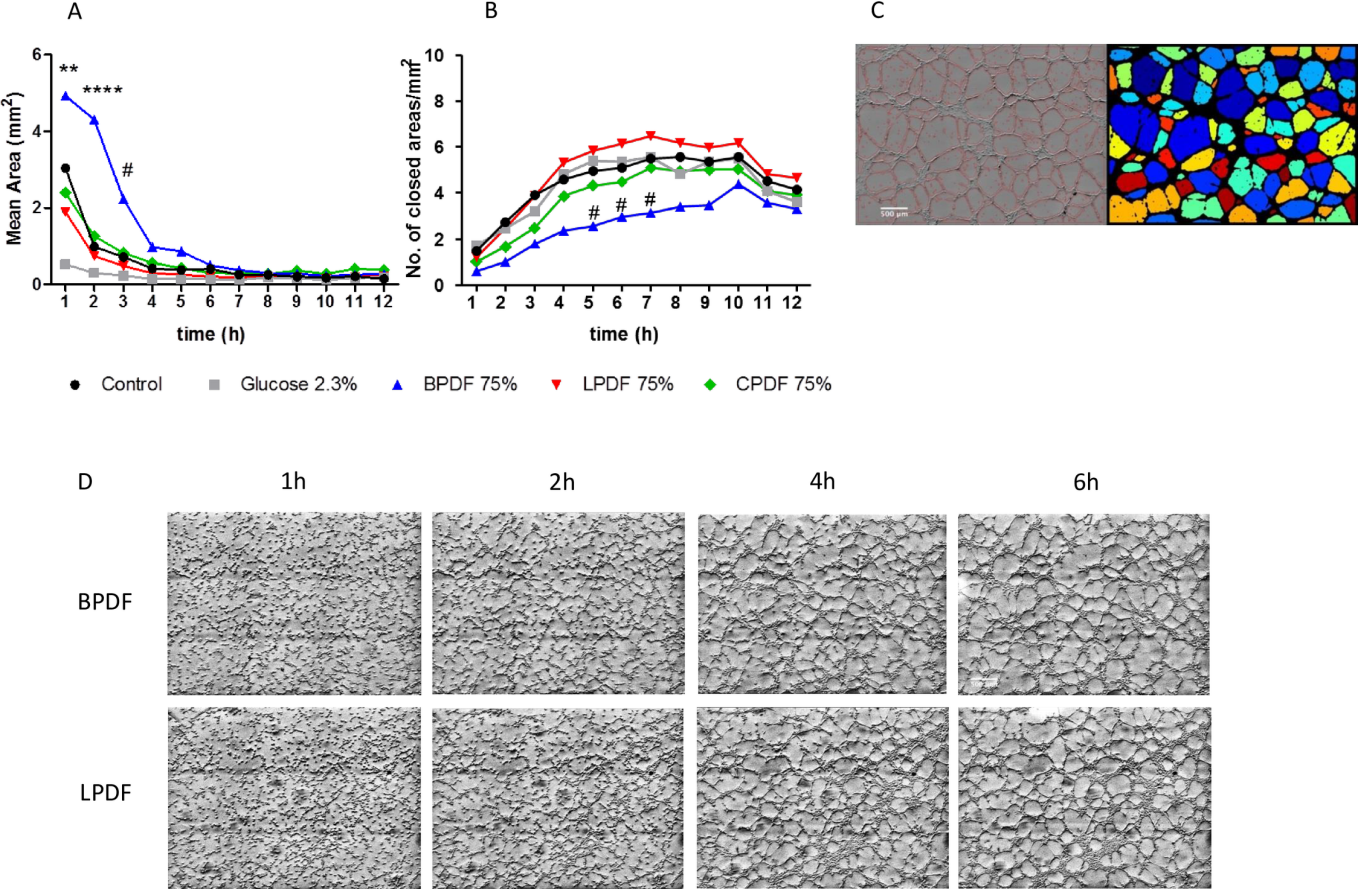
Tube formation assay. Angiogenic capacity analyzed by tube formation of human endothelial cells treated with different PD fluids. Automated analysis performed with CellProfiler Software showing (A) mean area and number of closed areas by endothelial cell branches (B) and (C) illustrative example of methodology (left image: red lines mark endothelial cells and their branches; right image: areas framed by endothelial cells are shown in different colors). Representative examples of the tubular network formed with BPDF and LPDF from 4 independent experiments performed in triplicates are given in (D). *p<0.05; **p<0.01; ****p<0.0001 for BPDF vs. all other; #p<0.05 BPDF vs. LPDF.

24 well plates each containing 285μl matrix gel (BD-Matrigel^®^) were incubated for 30 min. 5x10^4^ human umbilical vein endothelial cells per well were added in 500μl endothelial cell basal medium containing 2% fetal calf serum, 25 ng/ml VEGF-A and 25 ng/ml fibroblast growth factor-2 and incubated with different PD fluids for 24 hours. Hourly images (Zeiss Axiovert 200m microscope, Plan-NEOFLUAR 5x/0.15 lens) were acquired in bright field microscopy with oblique illumination and 7ms exposure time. To analyze a larger area, a mosaic of 3*3 pictures was taken of each well, covering at least 80% of the entire well. Each tile had an overlap of 20%, in order to appropriately merge tiles for analysis. Using Fiji [14], the single tiles were merged with the “Stitching” plugin. To accommodate unevenness in the matrigel surface and repositioning variations during the time-lapse 11-slice z-stack with 25μm spacing between slices were acquired. The images were projected into one plane with the “Extended Depth of Field” plugin [15] of Fiji. Images were analyzed by CellProfiler [16], based on the number and size of areas enclosed by endothelial tubes, which is robust to local cell density variations (Fig 1).

### Biopsy study

Peritoneal biopsies from age and PD vintage matched, peritonitis free children treated either with BPDF or LPDF (n = 8/group) were all obtained from the International Pediatric Peritoneal Biopsy Study (www.clinicaltrials.gov NCT01893710). Patients with organ failure beyond kidney diseases and systemic inflammatory disease were excluded. The study is conducted according to the principles expressed in the Declaration of Helsinki and was approved by the Ethic committee of the Medical Faculty, University of Heidelberg (S-487/2010). Informed written consent was obtained from all parents and patients. Patient age was 12.2–18.0 years (Table 1). Underlying diseases were glomerulonephritis (5), nephronophtisis (4), hypo-dysplastic kidneys (3), hemolytic-uremic syndrome (2), cystinosis (1), and Alport syndrome (1). Only one patient with LPDF and three patients treated with BPDF had residual urine output above 300 ml/m^2^ and day. Vessel analysis and immunohistochemical staining (anti-angiopoietin-1, 1:50, Sigma) were performed as previously described [17] and underwent automated quantification (Aperio®).

**Table 1:** 

	BPDF Mean (SD)	LPDF Mean (SD)	P value
Age (years)	15.1(2.0)	15.4 (1.8)	0.77
Body surface area (m^2^)	1.47 (0.28)	1.33 (0.30)	0.40
PD duration (months)	32.7 (25.3)	20.3 (25.4)	0.27
Dialytic glucose exposure (g/m^2^/day)	134 (30)	117 (41)	0.63
Hemoglobin (g/dl)	10.1 (1.8)	10.5 (1.7)	0.68
Serum calcium (mmol/l)	2.41 (0.11)	2.35 (0.24)	0.59
Serum phosphate (mmol/l)	1.52 (0.69)	2.03 (0.41)	0.14
Serum parathyroid hormone (pmol/l)	21.1 (14.2)	51.4 (40.1)	0.13
Serum creatinine (mg/dl)	9.85 (4.58)	11.83 (3.42)	0.45
Serum albumin (g/l)	36.9 (5.1)	31.6 (5.9)	0.19
Blood urea nitrogen (mg/dl)	62.6 (38.2)	59.1 (21.3)	0.84

BPDF = bicarbonate buffered, neutral pH PD fluid, LPDF = lactate buffered, neutral pH PD fluid

### Statistics

Data are from at least 4 independent sets of experiments, and all conditions were studied in triplicates in each of these experiments. Student t-test, one-way or two-way ANOVA corrected for multiple testing was used. Data are mean ± SD, p<0.05 was considered significant.

## Results

### Regulation of angiogenic cytokines by PDF

**Fig 2:**
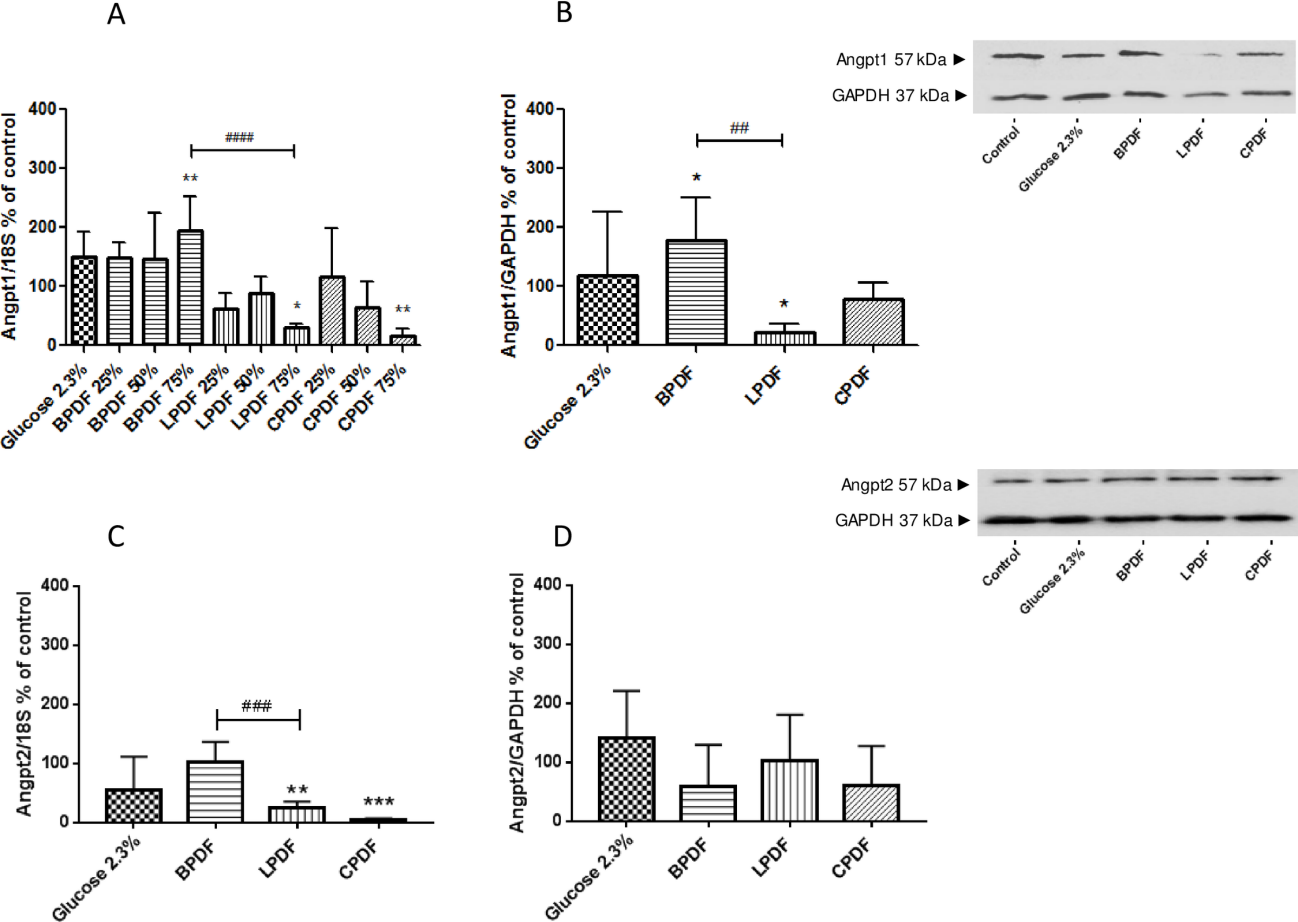
Angiopoietin1 and 2 mRNA-expression and protein abundance. Angiopoietin-1 (A) and angiopoietin-2 (C) mRNA expression and protein abundance (B, D) in human umbilical vein endothelial cells incubated with 75% PD fluid for 24h. *p<0.05, **p<0.01, ***p<0.001, ****p<0.0001 vs. Medium; ##p<0.01, ###p<0.001, ####p<0.0001 BPDF vs. LPDF.

Human umbilical vein endothelial cells were incubated with the different type of dialysate solutions diluted with medium free from fetal calf serum to dialysate concentrations of 25, 50 and 75% and with pure dialysate. At 75% dialysate concentration, angiopoietin-1 mRNA increased upon incubation with BPDF (194±59.2% of medium, p<0.01), was unchanged with 2.3% glucose (151±41%) and reduced with LPDF (29.9±6.6%; p<0.05) and CPDF (17.2±11.5% of medium; p<0.01; Fig 2). LDH release did not significantly differ between the PD treated groups and control. The effects were less pronounced with 25 and 50% PD fluid concentrations. 100% PD fluid resulted in cell toxicity, assessed visually and by LDH release. Subsequent experiments were therefore performed with 75% PD fluid.

Angiopoietin-1 protein abundance was increased with BPDF (179±71.5% of medium; p<0.05), reduced with LPDF (21.4±15%; p<0.05, p<0.01 vs. BPDF) and unchanged with CPDF (77.5±28%) and 2.3% glucose (117±93%; Fig 2).

Angiopoietin-2 mRNA was unchanged with BPDF (104±33%) and 2.3% glucose (55.2±56.5%) and reduced with LPDF (25±10.6%; p<0.01) and CPDF (5.1±2.1%; p<0.001). Angiopoietin-2 protein abundance was similar after incubation with the different PD fluids (BPDF 59.6±70.1%, LPDF 104±76.9%, CPDF 60.3±67.7%, 2.3% glucose 141±80.5% of medium control, all p = ns; Fig 2). Relative to medium Angiopoietin-1/Angiopoietin-2 protein ratio was threefold increased with BPDF, decreased to 20 and 50% with LPDF and glucose and unchanged with CPDF.

Other key cytokines involved in angiogenesis, VEGF-A, matrix metalloproteinase-1, heme oxygenase-1, fibroblast growth factor-2, aquaporin-1 and hypoxia inducible factor-1a were not differentially expressed in human umbilical vein endothelial cells upon BPDF and LPDF exposure. VEGF-A, matrix metalloproteinase-1 and heme oxygenase-1 mRNA increased with CPDF (218±117%; 175±93.8% and 1018±407% of medium p<0.0001/0.05/0.0001). VEGF-A protein was similar in all treatment groups.

Whole exome expression analyses in human peritoneal mesothelial cells [9] demonstrated significant regulation of heme oxygenase-1 by PD fluids (p<0.000001) but of no other gene known to be involved in angiogenesis. In a separate set of experiments using new human peritoneal mesothelial cells lines from 4 different donors, quantification of heme oxygenase-1, angiopoietin-1, angiopoietin-2, VEGF-A, matrix metalloproteinase-1, fibroblast growth factor-2 and hypoxia-inducible factor-1a by qRT-PCR demonstrated no difference following LPDF and BPDF treatment, respectively. Compared to medium, LPDF and CPDF increased heme oxygenase-1 (169±57.2%, 337±78.7%, p<0.05/0.0001), but not BPDF or 2.3% glucose (100±18%, 90.6±7.1%).

### Endothelial tube formation

To measure angiogenic capacity, the sprouting cell branches of human umbilical vein endothelial cells seeded on a matrigel were recognized by time lapse microscopy. The density of the formed network was calculated by measuring the number and size of enclosed areas. In a proangiogenic surrounding, a tighter network develops and therefore more closed areas with smaller areas can be found. PD fluids had a significant impact on the capillary network forming capacity of human endothelial cells, i.e. on the number of areas surrounded by human endothelial cells and on area size (both p<0.0001 for time and PD fluid type). Area size was higher during the first three hours of incubation with BPDF (4.9±4.8, 4.3±4.7, 2.2±2.4mm^2^ after 1, 2 and 3 hours) compared to LPDF (1.9±1.8, 0.7±0.7, 0.5±0.6mm^2^ p<0.0001/0.0001/0.05) and glucose (0.5±0.5, 0.3±0.2, 0.2±0.2mm^2^, p<0.0001/0.0001/0.05). After 1 and 2 hours of incubation area size was also lower with BPDF compared to CPDF (2.4±2.4, 1.3±1.3mm^2^; p<0.001/0.0001) and medium (3.0±4.3, 1.0±0.9mm^2^; p<0.01/0.0001). Numbers of endothelial tube surrounded areas was significantly lower after 5, 6 and 7 hours of incubation with BPDF compared to LPDF (5h: 2.8±2.6 vs. 6.3±4.3/mm^2^; 6h: 3.2±2.8 vs. 6.7±4.4/mm^2^; 7h: 3.4±2.7 vs. 7.0±4.6/mm^2^; all p<0.05, Fig 1).

### Receptor Tyrosine Kinase translocation

**Fig 3:**
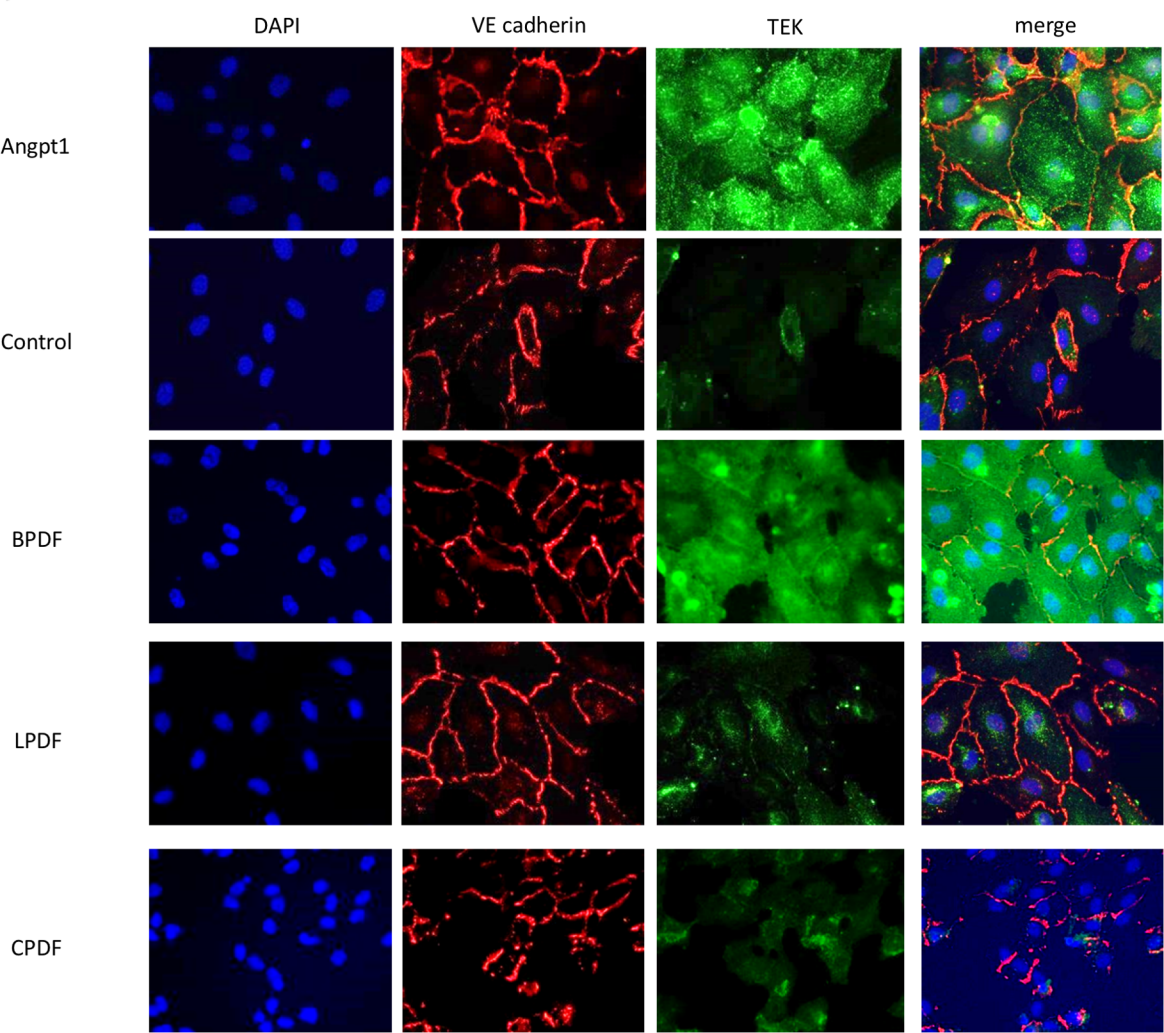
Translocation of Receptor Tyrosine Kinase receptor to cell-cell contacts. Immunofluorescence staining of human umbilical vein endothelial cells following incubation with recombinant human angiopoietin-1 (30min), dialysates and glucose (24h). Angiopoietin1 and bicarbonate buffered PD fluid promote translocation of Receptor Tyrosine Kinase to the vascular endothelial cadherin (VE cadherin) cell-cell contacts (orange overlap). Magnification 400x. Representative images are given.

Angiopoietin 1 is a Receptor Tyrosine Kinase agonist and promotes vascular stabilization via translocation of the receptor to cell-cell contacts [18]. We demonstrated the PD fluid buffer dependent effects on Receptor Tyrosine Kinase translocation by co-staining with the cell-cell contact marker vascular endothelial cadherin. In BPDF and recombinant human angiopoietin-1 treated endothelial cells, Receptor Tyrosine Kinase was more abundant than in LPDF, CPDF and glucose treated cells and co-localized with vascular endothelial cadherin on cell-cell contacts (Fig 3). LDH release was low and similar with all PD treatments, ruling out major unspecific PD fluid cell toxicity.

### Human peritoneal ex vivo findings

**Fig 4:**
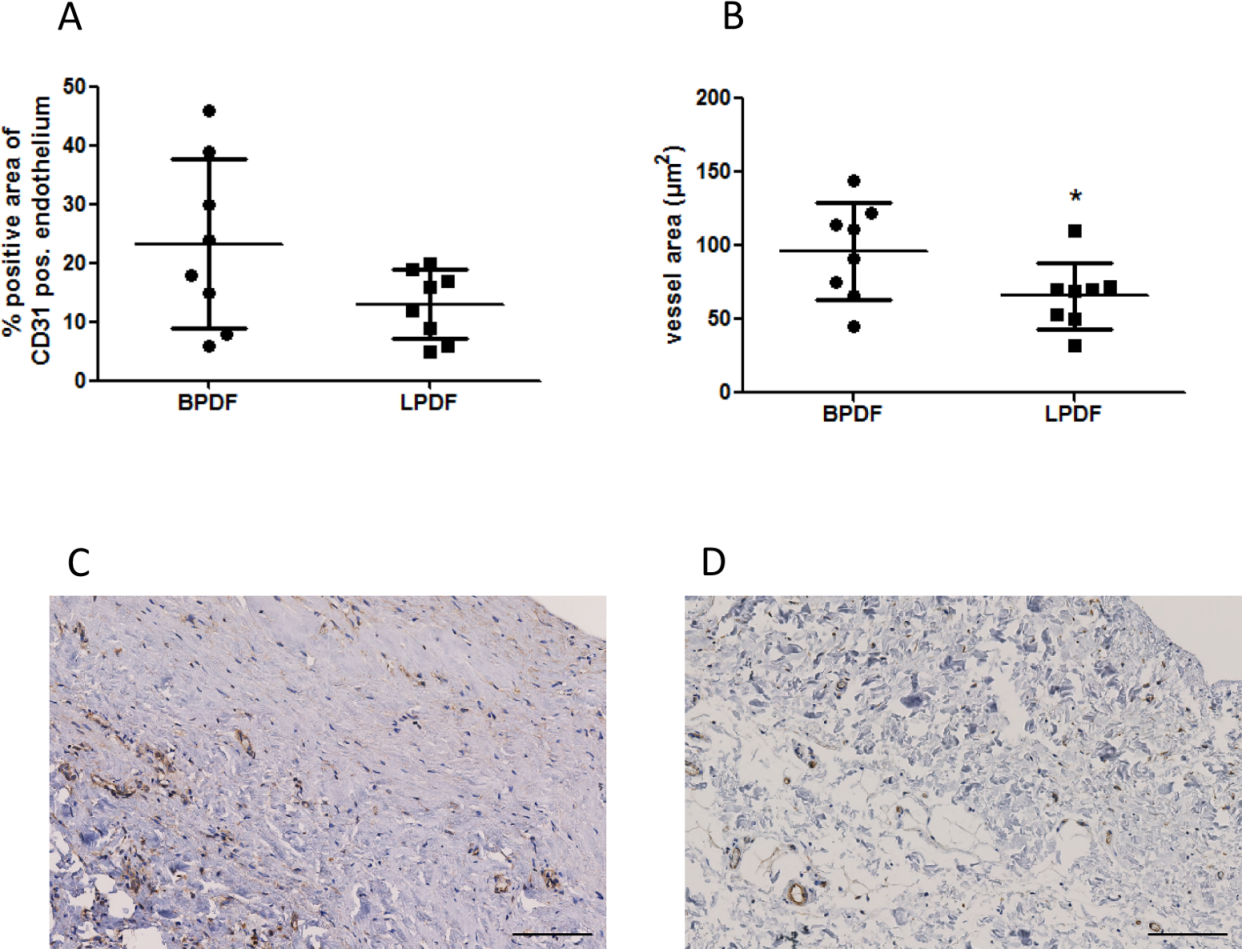
Vessel morphology and angiopoietin-1 abundance in peritoneal biopsies. Angiopoietin-1 abundance per CD31 positive endothelium (A) and cross sectional vessel area (B) in the peritoneum of children on PD with bicarbonate and lactate buffered PD fluid and low glucose degradation product content (n = 8/group; Aperio^®^ digital image analysis). Representative angiopoietin-1 staining with BPDF and LPDF are given in (C) and (D), scale bar 100μm. * p<0.05.

To validate experimental findings in the human PD setting, peritoneal tissue specimen from age, PD vintage and glucose exposure matched children treated with low glucose degradation product content PD fluid containing either lactate or bicarbonate buffer, were analyzed with regard to endothelial angiopoietin-1 abundance, vessel density and morphology. Peritoneal angiopoietin-1 abundance per CD31 positive endothelium was non-significantly higher in BPDF than in LPDF treated children (23±14 vs. 13±5.9, p = 0.08), vessel area increased (96±33 vs. 66±23μm^2^, p<0.05) (Fig 4). Vessel number was 59±19 vs. 111±151/mm peritoneum (p = ns).

## Discussion

Insufficient ultrafiltration rates result in chronic fluid overload, arterial hypertension and cardiovascular damage in patients on PD [19]. According to mathematical modeling [20], studies in rodents [21] and PD patients [1], overshooting angiogenesis is the primary pathomechanism of progressive ultrafiltration loss with time on PD. Increased peritoneal vessel density results in faster glucose absorption and dissipation of the osmotic gradient required for fluid removal [2]. The interstitial space is of little importance unless major submesothelial fibrosis develops [22]. We now provide data suggesting that bicarbonate compared to lactate buffered PD fluid increases endothelial angiopoietin1 synthesis and the angiopoietin-1/-2 ratio, promotes Receptor Tyrosine Kinase translocation to cell-cell contacts and thus shifts the balance from blood vessel formation towards vessel maturation [23]. *In vitro* endothelial vessel formation capacity is reduced. In age, PD vintage and glucose exposure matched children on chronic PD, peritoneal vessel area, an indicator of cell maturation [24] is higher with BPDF than with LPDF.

Endothelial cells are the key element in angiogenesis, proliferating and protruding into previously avascular tissue areas, where they coalesce to form a primitive tubular network [25]. Glucose and glucose degradation products, present at high concentrations in single chamber PD fluids, induce VEGF synthesis and secretion [26]. VEGF increases vascular permeability, allowing the extravasation of plasma proteins that form a primitive scaffold for migrating endothelial cells into the surrounding tissue matrix [27]. Angiopoietin-1 and angiopoietin-2 exert diverging functions during angiogenesis. Angiopoietin-1 reduces vascular permeability, protects against plasma leakage and promotes vessel maturation. It induces Receptor Tyrosine Kinase translocation to cell-cell contacts, which inhibits paracellular permeability and promotes vessel stabilization [28]. Angiopoietin-2 destabilizes vessels and promotes permeabilisation [23]. The VEGF and angiopoietin-1/-2 pathways and their receptors are almost exclusively endothelial cell specific [23]. We now provide data suggesting that depending on the PD buffer compound fluids with low glucose degradation products content regulate angiopoietin-1/angiopoietin-2 signaling. BPDF increases angiopoietin-1 and the angiopoietin-1/-2 ratio, and promotes Receptor Tyrosine Kinase translocation to the cell-cell contacts as demonstrated by co-staining with vascular endothelial-cadherin, i.e. shifts endothelial cells to a less angiogenic, maturated state, whereas LPDF exposure maintains a proangiogenic state [24]. In line with this, time integrated endothelial tube formation capacity, a well-established model of angiogenesis [29], is significantly lower with BPDF than with LPDF and CPDF. Thus, differences in the angiopoietin-1/angiopoietin-2 ratio of endothelial cells exposed to BPDF and LPDF translate into differences in endothelial vessel formation.

The role of the peritoneal mesothelial cells in the context of BPDF and LPDF induced angiogenesis is less clear. During PD mesothelial cells detach [1] or undergo epithelial to mesenchymal transition (EMT) into a myofibroblast like cell type, secreting profibrotic and proangiogenic cytokines [30]. The concept of epithelial to mesenchymal transition, however, has been questioned by lineage tracing technology, suggesting that EMT cells originate from fibroblasts rather than mesothelial cells [31]. We recently performed a whole exome analysis from human peritoneal mesothelial cells exposed to different PD fluids [9]. Heme oxygenase-1 expression but no other gene known to be involved in angiogenesis was regulated by PD fluids. Heme oxygenase-1 increases VEGF and angiogenesis [32]. We, however, did not observe differences in 7 cytokines involved in angiogenesis following incubation of HPMC with LPDF and BPDF. Thus, a role in PD buffer mediated peritoneal angiogenesis is unlikely.

Children are uniquely suited for analysis of specific PD related vascular changes, since -in contrast to adults- they are virtually free of age and lifestyle related tissue alterations. Out of a cohort of 57 children on chronic PD with purely bicarbonate or purely lactate buffered, fluids with low glucose degradation product content, who underwent a parietal peritoneal biopsy within a global effort of 37 pediatric centers, we selected small, but very well matched cohorts, comparable regarding age, PD vintage, and PD glucose exposure, and without a recent history of peritonitis. Thus, potential possible bias of factors known to influence peritoneal vascularization was minimized [1, 2]. According to the International Pediatric PD Network (www.pedpd.org) more than two third of children have still been treated with single chamber fluids with high glucose degradation product content, 13% with a solution containing both lactate and bicarbonate buffer, facts which further impede a larger scale histomorphometric analysis. Vessel morphology was quantified by automated image analyses, precluding an investigator related bias. Mean endothelial angiopoietin-1 abundance was 55%, albeit not reaching statistical significance level, vessel area was 30% higher with BPDF than with LPDF. Cross sectional area is higher in maturated than in growing vessels [24]. Vessel number was 47% lower with BPDF, albeit without reaching significance in the small group of patients. Thus our human *ex vivo* findings are in line with the experimental findings of a significant role of the PD fluid buffer, lactate and bicarbonate, in angiogenesis. They moreover suggest a potential molecular mechanism for the observed better preservation of ultrafiltration capacity in children treated with BPDF as compared to LPDF [7]. In view of the limited efficacy of PD and the progressive alteration of PD membrane function with chronic PD [33] and the resulting cardiovascular sequelae [19], these findings are of great interest. On the other hand, the limited number of patients analyzed in this study and in the only clinical trial thus far evaluating the role of the buffer compound of PD fluids with low glucose degradation product content [7], does not allow drawing firm conclusions on clinical practice of PD fluid choice. Larger patient cohorts have to be studied over extended periods of time, at best in randomized controlled trials,

In conclusion, bicarbonate containing PD fluids with low glucose degradation product content promote vessel maturation via upregulation of angiopoietin-1 and translocation of Receptor Tyrosine Kinase to cell-cell contacts, whereas the respective lactate containing PD fluid maintains a proangiogenic state of endothelial cells. These mechanisms may impact on peritoneal ultrafiltration capacity in patients undergoing chronic PD with different PD fluid buffer compound.
